# Editorial: Neurotransmitters and Emotions, Volume II

**DOI:** 10.3389/fpsyg.2022.920702

**Published:** 2022-05-27

**Authors:** Fushun Wang, Jiongjiong Yang, Fang Pan, Roger C. Ho, Jason H. Huang

**Affiliations:** ^1^Institute of Brain and Psychological Science, Sichuan Normal University, Chengdu, China; ^2^Department of Psychology, Beijing University, Beijing, China; ^3^Department of Psychology, Shandong University, Jinan, China; ^4^Department of Psychology, National University of Singapore, Singapore, Singapore; ^5^Department of Neurosurgery, Baylor Scott & White Health Center, Temple, TX, United States

**Keywords:** emotion, neurotransmitter, neuromodulator, basic emotions, dopamine, monoamine

In spite of its central importance for human beings, emotion is still one of the least studied subjects among the life sciences (Gu et al., 2016). Even though the last century has witnessed many progresses in emotional studies, psychological analyses have not resolved many of the core issues surrounding emotions (Xu et al., 2022), such as the number of basic emotions, and the definition of basic emotions. Although there has been no shortage of psychological research on these debates, for instance, there is little agreement about how many emotions are “basic,” which emotions are “basic,” and why they are “basic” (Liang et al., 2021). Anyway, studies of the neural basis of emotions in the brain might resolve these questions. Indeed, studies of the neural basis of emotion have a long history and are still an active subfield within experimental and theoretical research (Gu et al., 2019).

Many scientists started to identify the neural structures that are responsible for particular emotions in the mid-twentieth century, and found that the limbic system might be the brain locus for emtions, and called Papez circuit the emotional brain. These pioneering work outlined the neural anatomical basis of emotion, and helped locate many important structures involved in basic emotions, such as the amygdala, which might be the place for fearful emotions (Xu et al., 2022). However, these investigations stopped with the development of behaviorism, which suggested that emotion is not a kind of behavior that can be objectively studied in the laboratory, because of its introspection nature. LeDoux (2000) proposed that: “The field of neuroscience has, after a long period of looking the other way, again embraced emotion as an important research area” (LeDoux, 2000).

The rigorous investigation of the neural basis of emotions returned at the end of the twentieth century, however, many recent studies found that the brain structures for basic emotions are usually mixed (Lindquist et al., 2012). These inconsistent studies on the neural basis of basic emotions induced more debates in the field of emotions (Lindquist et al., 2013), even some people suspect the theory of basic emotions. Basic emotions are suggested to be evolved for fundamental life tasks, which are present for not only human beings, but also for all animal kinds. Darwin suggested that the phylogenetically lower animals, such as insects, also have basic emotions. However, the brain structure of the vertebrate brain is quite different from that of an insect. There is no similar limbic system in the invertebrate brain, let alone the amygdala, but they do have monoamine neuromodulators (Gu et al., 2018a). Thus, we suggest that the monoamine neurotransmitters might be the neural basis for emotion.

The neurotransmitter approach for emotion hypothesizes that the emotions are not special functions for one small brain area, instead, they are due to widely projected neuromodulators (Gu et al., 2016). This might really be the case that the monoamine neuromodulators, including dopamine, norepinephrine, and serotonin all project widely to almost the whole brain (Gu et al., 2019). The monoamine neuromodulators are still the targets for the first-line antidepressant drugs (Gu et al., 2018a), after decades after the discovery of drugs for affective disorders. In addition, the etiology of many affective disorders are related to the monoamine neuromodulators (Lovheim, 2012). Thus, monoamine (DA, NE, and 5-HT) has been regarded as the emotional substrate ever since their discovery. In addition, many recent emotional theories are introduced based on these three monoamines (He et al., 2021), including our three primary color model of basic emotions (Gu et al., 2018b), which proposed that there are only three kinds of basic emotions: disgust, joy, and fear for all the animal kinds. The three basic emotions depend on the monoamine neuromodulators, 5-HT is the substrate for disgust, DA is the substrate for joy; and NE is the neural basis for sympathetic function, and for fear (anger) emotions, for “fight or flight” behaviors (Liang et al., 2021).

The monoamine neuromodulators are the primary neural basis for emotions, however, there are many other chemicals in the brain or endocrine systems that are related to emotions, such as corticotropin-releasing hormone (CRH) or cortisone, oxytocin, or sex hormone. Oxytocin has been recently found to be the neuromodulator for love and attachment (Aguilar-Raab et al., 2019). The nasal application of oxytocin can induce positive emotions. In addition, the hormones from the hypothalamic-pituitary-adrenal (HPA) axis, such as CRH, ACTH (adrenocorticotropic hormone), and cortisol, have been proved to be involved in the stress process. Furthermore, the sex hormone progesterone has been proved to affect disgust (Simeon et al., 2007). In all, many neurotransmitters have been suggested to be involved in emotions. In this special topic, we invited recent studies that focus on the emotional functions of neurotransmitters, and have got 15 submissions. After half a year's critical peer review, we have got 4 papers accepted.

In the paper titled “*Autonomic Nervous System Response Patterns of Test-Anxious Individuals to Evaluative Stress*,” Bian et al. investigated the role of autonomous nervous system in test anxiety, and found that high test-anxious individuals display an increased sympathetic nervous system activity, with high norepinephrine activity together with decreased parasympathetic nervous system activity in response to stress. While low anxious individuals display an increased sympathetic nervous system activity with a stable parasympathetic nervous system.

In the paper titled “*Emotionality vs. Other Biobehavioral Traits: A Look at Neurochemical Biomarkers for Their Differentiation*,” Trofimova et al. reviewed differential contributions of multiple neurochemical systems to temperament traits related and those that are unrelated to emotionality. They especially focused on the differential contribution of hypothalamic-pituitary hormones and opioid neuropeptides implicated in both emotional and non-emotional regulation. They suggest that the mu-opioid receptor system is involved in emotional valence while the kappa-opioid system is involved in arousal or behavioral alertness. In addition, they also suggested that empathy is based on (higher) oxytocin, reciprocally coupled with vasopressin and (lower) testosterone.

In the paper titled “*Roles of Anxiety and Depression in Predicting Cardiovascular Disease Among Patients With Type 2 Diabetes Mellitus: A Machine Learning Approach*,” the authors Chu et al. tried to investigate the underlying neural mechanisms for the emotional problems that are related to cardiovascular diseases in a bio-psycho-social way.

In the paper titled “*Affective Face Processing Modified by Different Taste*,” the author Liang et al. reported their studies about the cross-modal interaction between taste and emotional face search using two tastes (sweet and acid) to investigate the cross-modal interaction between taste and emotional face search and their underlying mechanisms.

Collectively, these studies demonstrate that neurotransmitters play an important role in emotion. We hope that this special issue will stimulate interest in the field of basic emotion.

## Author Contributions

FW, RH, FP, JY, and JH wrote the paper. All authors contributed to the article and approved the submitted version.

## Funding

The paper was supported by a grant from Foundation of Humanities and Arts from the Ministry of Education in China (19YJAZH083).

## Conflict of Interest

The authors declare that the research was conducted in the absence of any commercial or financial relationships that could be construed as a potential conflict of interest.

## Publisher's Note

All claims expressed in this article are solely those of the authors and do not necessarily represent those of their affiliated organizations, or those of the publisher, the editors and the reviewers. Any product that may be evaluated in this article, or claim that may be made by its manufacturer, is not guaranteed or endorsed by the publisher.
